# A dual role of miR-22 modulated by RelA/p65 in resensitizing fulvestrant-resistant breast cancer cells to fulvestrant by targeting *FOXP1* and *HDAC4* and constitutive acetylation of p53 at Lys382

**DOI:** 10.1038/s41389-018-0063-5

**Published:** 2018-07-30

**Authors:** Bo Wang, Dongping Li, Jody Filkowski, Rocio Rodriguez-Juarez, Quinn Storozynsky, Megan Malach, Emily Carpenter, Olga Kovalchuk

**Affiliations:** Department of Biological Sciences, University of Lethbridge, Lethbridge, AB Canada

## Abstract

Antiestrogen resistance is a major challenge encountered during the treatment of estrogen receptor alpha positive (ERα^+^) breast cancer. A better understanding of signaling pathways and downstream transcription factors and their targets may identify key molecules that can overcome antiestrogen resistance in breast cancer. An aberrant expression of miR-22 has been demonstrated in breast cancer; however, its contribution to breast cancer resistance to fulvestrant, an antiestrogen drug, remains unknown. In this study, we demonstrated a moderate elevation in miR-22 expression in the 182^R^-6 fulvestrant-resistant breast cancer line we used as a model system, and this elevation was positively correlated with the expression of the miRNA biogenesis enzymes AGO2 and Dicer. The level of phosphorylated HER2/neu at Tyr877 was also upregulated in these cells, whereas the level of RelA/p65 phosphorylated at Ser536 (p-p65) was downregulated. Knockdown of HER2/neu led to an induction of p-p65 and a reduction in miR-22 levels. Luciferase assays identified two NF-κB binding motifs in the miR-22 promoter that contributed to transcriptional repression of miR-22. Activation of RelA/p65, triggered by LPS, attenuated miR-22 expression, but this expression was restored by sc-514, a selective IKKβ inhibitor. Inhibition of miR-22 suppressed cell proliferation, induced apoptosis and caused cell cycle S-phase arrest, whereas enhancing expression of p21^Cip1/Waf1^ and p27^Kip1^. Surprisingly, ectopic expression of miR-22 also suppressed cell proliferation, induced apoptosis, caused S-phase arrest, and promoted the expression of p21^Cip1/Waf1^ and p27^Kip1^. Ectopic overexpression of miR-22 repressed the expression of FOXP1 and HDAC4, leading to a marked induction of acetylation of HDAC4 target histones. Conversely, inhibition of miR-22 promoted the expression of both FOXP1 and HDAC4, without the expected attenuation of histone acetylation. Instead, p53 acetylation at lysine 382 was unexpectedly upregulated. Taken together, our findings demonstrated, for the first time, that HER2 activation dephosphorylates RelA/p65 at Ser536. This dephosphoryalted p65 may be pivotal in transactivation of miR-22. Both increased and decreased miR-22 expression cause resensitization of fulvestrant-resistant breast cancer cells to fulvestrant. HER2/NF-κB (p65)/miR-22/HDAC4/p21 and HER2/NF-κB (p65)/miR-22/Ac-p53/p21 signaling circuits may therefore confer this dual role on miR-22 through constitutive transactivation of p21.

## Introduction

Breast cancer is one of the most common malignancies that threaten women’s health worldwide and is the second leading cause of cancer-related deaths in North American women (GLOBOCAN 2012, http://globocan.iarc.fr/Pages/fact_sheets_population.aspx). Most breast cancers express estrogen receptor alpha (ERα)^1^, a member of the steroid/thyroid receptor superfamily that primarily mediates the biological functions of estrogen through binding^2^. Estrogen/ERα signaling is a known contributor to the proliferation of ERα-positive breast cancers^3^, so endocrine therapy (also known as hormonal therapy) targeting the estrogen/ERα signaling is now well established as an efficient adjuvant treatment for patients with ERα-positive breast cancers^4^. The most commonly used endocrine therapeutic agents that target ERα-positive breast cancers include ER modulators (e.g., tamoxifen, which selectively antagonizes ERα function), ER downregulators (e.g., fulvestrant, also known as ICI 182,780 and faslodex, which selectively downregulates ERα), and aromatase inhibitors (e.g., letrozole and anastrozole, which repress estrogen production by attenuating aromatase activity)^3,5^. A large body of evidence from both basic and clinical studies has now demonstrated the efficacy of tamoxifen and fulvestrant in patients with breast cancer^6–9^. Furthermore, comparison with 5-year exposure has confirmed that continuing tamoxifen treatment for 10 years further reduced the risk of disease recurrence and mortality in a randomized trial of patients with ER-positive breast cancer^10^. However, long-term exposure may eventually lead to acquisition of drug resistance^11–13^, which is often the cause of treatment failure and is now becoming a serious clinical problem in hormonal therapy. The mechanisms underlying this antiestrogen resistance are not yet completely understood.

In the last two decades, one important advance in bioscience has been the discovery of microRNAs (miRNAs/miRs), the key players in post-transcriptional regulation of gene expression. The microRNAs are the most abundant class of small non-coding RNAs, and extensive studies have demonstrated that they exert either oncogenic or tumor-suppressive effect on cells by negatively regulating gene expression through either translational repression or mRNA degradation^14,15^. Overall, only 30–60% of protein-coding genes are thought to be targets of miRNAs, but these may explain all aspects of the diverse physiologic and pathologic functions of miRNAs^16–18^, including drug resistance. Of the known miRNAs, the best defined is miR-221, which plays a pivotal role in the development of anticancer drug resistance in many human malignancies, including fulvestrant and tamoxifen resistance in breast cancer^19,20^.

Accumulating evidence now indicates that deregulation of miR-22 contributes to several hallmarks of breast cancer^21,22^ and that miR-22 overexpression resensitizes paclitaxel-resistant colon cancer cells to paclitaxel^23^. However, the role of miR-22 in fulvestrant resistance in breast cancer cells remains unknown. In the present study, we examined the contribution of miR-22 to the fulvestrant resistance of breast cancer and the potential transcriptional control of this miRNA by NF-kB (RelA/p65, p-p65). Surprisingly, we found that ectopic expression and knockdown of miR-22 both enhanced the fulvestrant sensitivity of the fulvestrant-resistant 182^R^-6 breast cancer cell line. This enhancement occurred through the upregulation of p21^Cip1/Waf1^ and p27^Kip1^ by targeting the transcriptional repressor/corepressor *FOXP1* and *HDAC4*. We also discovered that the upregulation of phosphorylated HER2/neu (Tyr877) in 182^R^-6 cells was negatively correlated with the phosphorylation of RelA/p65 (Ser536). Knockdown of HER2/neu caused an induction in p-p65 and a reduction in miR-22. Luciferase assays identified two NF-κB binding motifs in the miR-22 promoter that contributed to the transcriptional repression of miR-22. Our results revealed that HER2/RelA (p65)/miR-22 signaling plays a crucial role in resensitizing fulvestrant-resistant 182R-6 cells to fulvestrant by upregulating p21^Cip1/Waf1^ and/or p27^Kip1^ by targeting of the transcriptional repressor/corepressor FOXP1 and HDAC4 and constitutive acetylation of p53 at Lys382.

## Results

### Induction of miR-22 in fulvestrant-resistant breast cancer cells

**Fig. 1:**
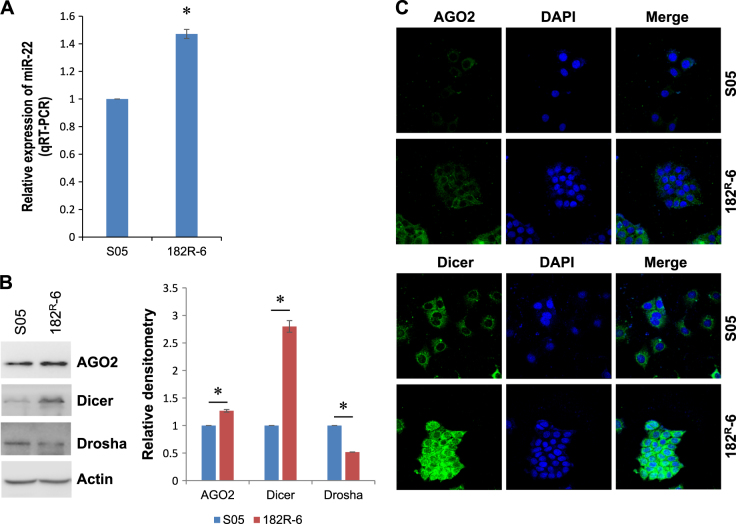
The moderate induction of miR-22 may be attributed to the overexpression of AGO2 and Dicer in fulvestrant-resistant breast cancer 182^R^-6 cells. **a** Total RNA isolated from the 182^R^-6 cell line and its parental S05 cell line was subjected to qRT-PCR analysis using a primer set for hsa-miR-22. RNU6-2 was used as a reference gene to normalize miR-22 expression. **b** Whole cellular lysates prepared from S05 and 182^R^-6 cells were subjected to western blot analysis using antibodies to either AGO2 or Dicer or Drosha; actin served as a loading control. **c** S05 and 182^R^-6 cells grown to 60–75% confluency on glass coverslips were fixed with 4% paraformaldehyde and permeabilized with ice-cold methanol. Immunofluorescence staining was performed using antibodies to either AGO2 or Dicer, as described in the “Methods” section. * indicates *p* < 0.05

To identify miRNAs with possible involvement in antiestrogen-resistant breast cancer, we performed a global miRNA microarray to screen the miRNAs that were differentially expressed between the parental line and a fulvestrant- or tamoxifen-resistant line. Global miRNA profiling using two reference genes (RNU24 and RNU38B) identified four differentially expressed miRNAs that showed identical changes in both fulvestrant-resistant and tamoxifen-resistant lines, including upregulation of miR-21, miR-22, and miR-23a and downregulation of miR-27b (Supplementary Fig. S1). However, only the upregulation of miR-22 was further validated in both the fulvestrant-resistant (Fig. 1a) and tamoxifen-resistant (data not shown) lines, using quantitative real-time RT-PCR (qRT-PCR) and RNU6B as a reference gene (this differed from the genes used in the miRNA microarray).

The previously indicated efficacy of fulvestrant for both advanced and tamoxifen-resistant breast cancers^24^ made miR-22 in fulvestrant resistance of particular interest in the present study. We looked at the contribution of argonaute 2 (AGO2), Dicer, and Drosha to the biogenesis of miR-22. Western blot analysis showed an overexpression of AGO2 and Dicer in fulvestrant-resistant 182^R^-6 cells (Fig. 1b), and this overexpression was confirmed by immunofluorescence (Fig. 1c). By contrast, Drosha expression was slightly decreased (Fig. 1b). These results suggest an involvement of AGO2 and Dicer in the induction of miR-22 in 182^R^-6 cells.

### Altered expression of miR-22 sensitizes 182^R^-6 cells to fulvestrant, induces S-phase arrest and triggers apoptosis by upregulating p21^Cip1/Waf1^ and p27^Kip1^

**Fig. 2:**
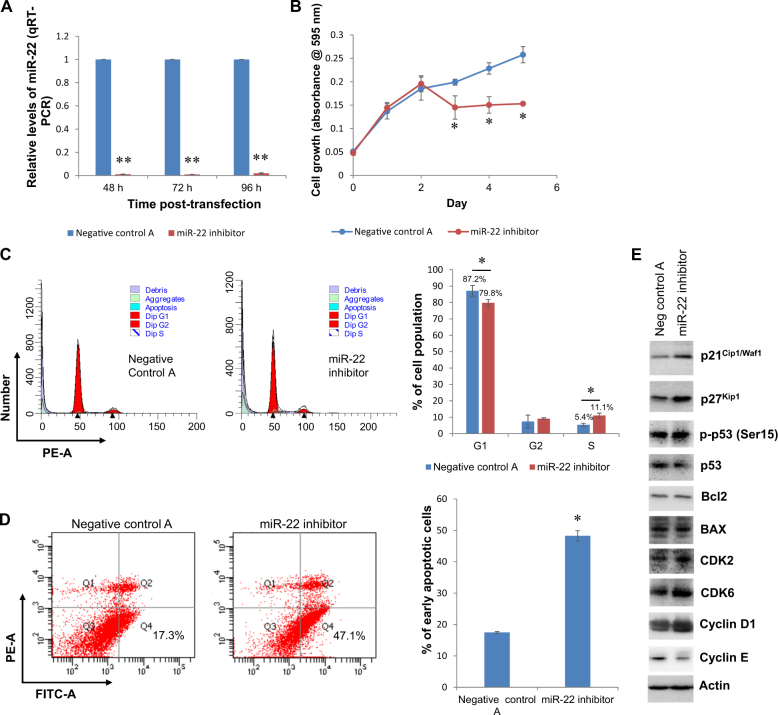
Inhibition of miR-22 suppressed 182^R^-6 cell proliferation, induced apoptosis, and caused cell cycle S-phase arrest via overexpression of p21 and p27. **a** 182^R^-6 cells grown to 80% confluency in a DMEM/F12 medium supplemented with 0.1 μM fulvestrant were transfected with either 50 nM miR-22 inhibitor or negative control A. At 48, 72, and 96 h after transfection, total RNA was isolated and subjected to qRT-PCR analysis using a primer set of hsa-miR-22; RNU6-2 was used as a reference gene to normalize miR-22 expression. **b** At  24 h after transfection, the 182^R^-6 cells were reseeded in 96-well plates and the MTT assay was performed as described in the “Methods” section. **c** At 96 h after transfection, the 182^R^-6 cells were harvested, and cell cycle analysis was performed as described in the “Methods” section. **d** At 96 h after transfection, the 182R-6 cells were harvested, and apoptosis analysis was performed as described in the “Methods” section. **e** At 96 h after transfection, whole-cell lysates were prepared and subjected to western blot analysis using the indicated antibodies, as described in the “Methods” section. Actin served as a loading control. * indicates *p* < 0.05. ** indicates *p* < 0.001

**Fig. 3:**
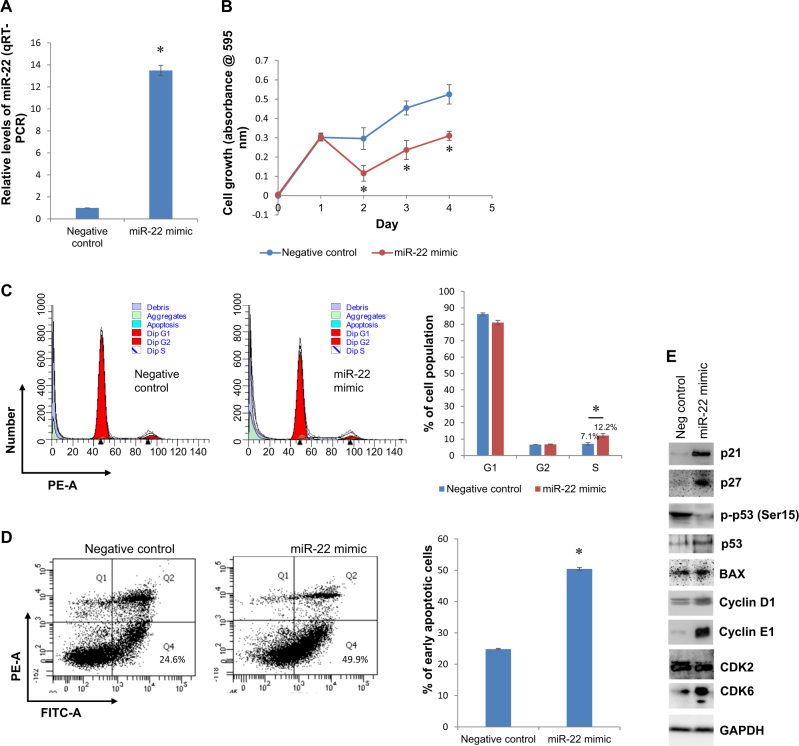
Enforced overexpression of miR-22 repressed 182^R^-6 cell proliferation, induced apoptosis, and caused cell cycle S-phase arrest via overexpression of p21 and p27. **a** 182^R^-6 cells grown to 80% confluency in a DMEM/F12 medium supplemented with 0.1 μM fulvestrant were transfected with either 40 nM miR-22 mimic or AllStars negative control siRNA. At 72 h after transfection, total RNA was isolated and subjected to qRT-PCR analysis using a primer set of hsa-miR-22; RNU6-2 was used as a reference gene to normalize miR-22 expression. **b** At  24 h after transfection, the 182^R^-6 cells were reseeded in 96-well plates, and the MTT assay was performed as described in the “Methods” section. **c** At 72 h after transfection, the 182^R^-6 cells were harvested, and cell cycle analysis was performed as described in the “Methods” section. **d** At 72 h after transfection, the 182^R^-6 cells were harvested, and apoptosis analysis was performed as described in the “Methods” section. **e** At 72 h after transfection, whole cell lysates were prepared and subjected to western blot analysis using the indicated antibodies, as described in the “Methods” section. Actin served as a loading control. * indicates *p* < 0.05

We explored the role miR-22 in fulvestrant-resistant 182^R^-6 cells using transient transfection. Inhibition of miR-22 with a miR-22 inhibitor significantly suppressed 182^R^-6 cell proliferation (Fig. 2a, b, *p* < 0.05), induced apoptosis and caused cell cycle S-phase arrest (Fig. 2c, d). Western blot analysis indicated upregulated expression of both the cell cycle inhibitors p21^Cip1/Waf1^ and p27^Kip1^ and the cell cycle drivers cyclin D1, CDK2, and CDK6 in 182^R^-6 cells in response to miR-22 inhibitor treatment (Fig. 2e). The miR-22 inhibitor reduced cyclin E expression, but it had no effect on the expression of phosphorylated p53 (Ser15, p-p53), Bcl2, and BAX (Fig. 2e). The biological function of miR-22 was further validated by transfecting 182^R^-6 cells with a miR-22 mimic. Surprisingly, the ectopic expression of miR-22 also markedly inhibited 182^R^-6 cell proliferation (Fig. 3a, b, *p* < 0.05), induced apoptosis and caused S-phase arrest (Fig. 3c, d). Interestingly, western blot analysis showed a profound elevation of expression of p21^Cip1/Waf1^, p27^Kip1^, cyclin E1, and CDK6 in 182^R^-6 cells in response to the miR-22 mimic (Fig. 3e). Treatment with the miR-22 mimic had no effect on the expression of BAX, CDK2, and cyclin D1, although it caused a reduction in p-p53 (Fig. 3e). Furthermore, p21^Cip1/Waf1^ expression was downregulated in fulvestrant-resistant 182^R^-6 breast cancer cells (supplementary Fig. S2). Taken together, the results indicated that 182^R^-6 cells were sensitized to fulvestrant by either knockdown or ectopic expression of miR-22 due to inhibition of proliferation and induction of S-phase arrest and apoptosis through upregulation of p21^Cip1/Waf1^ and p27^Kip1^.

### miR-22-3p promotes p21^Cip1/Waf1^ and/or p27^Kip1^ expression by directly targeting transcriptional repressors

**Fig. 4:**
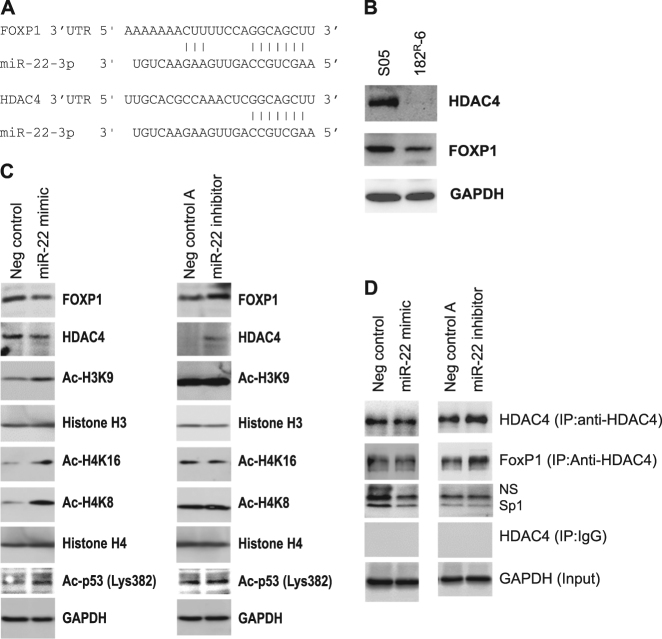
Targeting of HDAC4 and FOXP1 by miR-22 and constitutive acetylation of p53 may contribute to the overexpression of p21 and/or p27. **a** Diagram of the “seed” sequence for miR-22 targeting of HDAC4 and FOXP1. **b** Whole cell lysates prepared from S05 and 182^R^-6 cells were subjected to western blot analysis using antibodies to either HDAC4 or FOXP1. GAPDH served as a loading control. **c** 182^R^-6 cells transfected with either 40 nM miR-22 mimic or AllStars negative control siRNA or 50 nM miR-22 inhibitor or negative control were incubated for either 72 or 96 h. Whole cellular lysates were prepared and subjected to western blot analysis using the indicated antibodies. GAPDH served as a loading control. **d** 182^R^-6 cells transfected with either 40 nM miR-22 mimic or AllStars negative control siRNA or 50 nM miR-22 inhibitor or negative control were incubated for either 72 or 96 h. Whole cell lysates were prepared, and the immunoprecipitation and western blot analysis were performed as described in the “Methods” section

Our next objective was to determine how miR-22 regulates p21^Cip1/Waf1^ and p27^Kip1^ expression. Our bioinformatics analysis identified a potential binding motif between miR-22 and p21^Cip1/Waf1^ 3′-UTR (Supplementary Fig. S3a); therefore, we generated a plasmid reporter to explore whether miR-22 could directly control p21^Cip1/Waf1^ expression. The luciferase assay showed no significant interaction between miR-22 and the p21^Cip1/Waf1^ 3′-UTR (Supplementary Fig. S3b), suggesting that p21^Cip1/Waf1^ may not be a direct target of miR-22. We therefore considered the possible involvement of the transcriptional repressors forkhead box protein P1 (FOXP1) and histone deacetylase 4 (HDAC4), which are known to repress transcription of both p21^Cip1/Waf1^ and p27^Kip1^^25–29^, in the miR-22-mediated transcriptional activation of these two genes. Bioinformatics analysis identified a common “seed sequence” for miR-22 targeting both FOXP1 and HDAC4 (Fig. 4a), and the target sites in 3′-UTR of both FOXP1 and HDAC4 are evolutionarily highly conserved (supplementary Fig. S4), implicating miR-22 in the regulation of FOXP1 and HDAC4 expression. We tested this hypothesis by measuring the levels of both FOXP1 and HDAC4 in 182^R^-6 cells. As expected, we saw a decreased expression of FOXP1 and HDAC4 in 182^R^-6 cells compared with its parental line (Fig. 4b), and the expression was negatively correlated with miR-22 levels (Fig. 1a). We further confirmed these results by transfecting 182^R^-6 cells with either a miR-22 mimic or an inhibitor. Western blot analysis showed that ectopic overexpression of miR-22 repressed the expression of FOXP1 and HDAC4, leading to a marked induction of acetylation of four HDAC4 target proteins, Ac-H3K9, Ac-H4K8, Ac-H4K16, and Ac-p53 (Lys382) (Fig. 4c). Conversely, inhibition of miR-22 by an inhibitor promoted the expression of both FOXP1 and HDAC4 in 182^R^-6 cells (Fig. 4c). However, we did not see the expected attenuation of histone acetylation (Fig. 4c), but the level of acetylated p53 (acetylation at lysine 382) was upregulated (Fig. 4c). Immunoprecipitation using HDAC4 monoclonal antibody indicated that the ectopic expression of miR-22 attenuated the transcriptional repressor complex that would inhibit Sp1-driven transcription (Fig. 4d). The inhibition of miR-22 profoundly increased the amount of both FOXP1 and HDAC4 in the complex, but Sp1 was not elevated correspondingly (Fig. 4d).

A demethylase, the TET oncogene family member 2 (TET2), has recently been identified as a direct target of miR-22^21^. Therefore, we measured the potential effect of miR-22 on TET2 expression in the 182^R^-6 cell line. However, western blot analysis indicated no influence on TET2 levels by either enforced overexpression or inhibition of miR-22 (supplementary Fig. S5). Taken together, these results suggest that miR-22 may directly regulate the expression of the transcriptional repressors FOXP1 and HDAC4 and give rise to alterations in histone and p53 acetylation, which may consequently affect the expression of p21^Cip1/Waf1^ and p27^Kip1^.

### RelA/p65 Ser536 phosphorylation contributes to transcriptional repression of miR-22

**Fig. 5:**
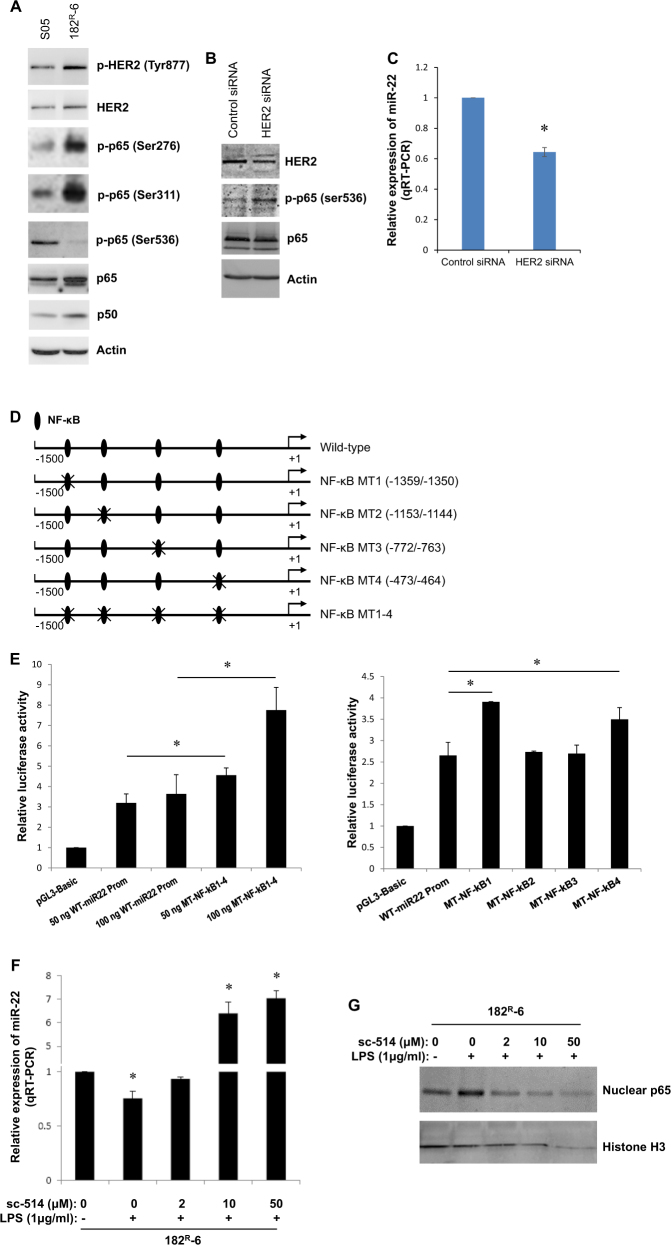
The HER2/p65 dephosphorylation (Ser536) axis negatively modulates miR-22 expression. **a** Whole cell lysates prepared from S05 and 182^R^-6 cells were subjected to western blot analysis using the indicated antibodies; actin served as a loading control. **b** 182^R^-6 cells were transiently transfected with either 100 nM Neu siRNA (pool of 3–5 target-specific 19–25 nucleotide sequences in length) or control siRNA-A. At 72 h after transfection, whole cell lysates were prepared, and the western blot analysis was performed using the indicated antibodies, as described in the “Methods” section. Actin served as a loading control. **c** 182^R^-6 cell were transfected with either 100 nM Neu siRNA or control siRNA-A. At 72 h after transfection, the total RNA was isolated, and qRT-PCR was performed using a primer set for hsa-miR-22. RNU6-2 was used as a reference gene to normalize the miR-22 expression. **d** Diagram of wild-type and mutant miR-22 promoter constructs. **e** HEK293 cells grown to 90% confluency were transiently transfected with either 50 or 100 ng reporter plasmid in combination with 0.5 μg GFP-RelA and 5 ng pRL-TK plasmid. At 24 h after transfection, the luciferase activity was measured as described in the “Methods” section. **f**, **g** 182^R^-6 cells grown to 80% confluency were exposed to the indicated concentrations of the IKK2 inhibitor sc-514. 1 h after exposure, the cells were incubated with the indicated concentration of LPS. At 24 h after treatment, total RNA was isolated and subjected to qRT-PCR analysis for miR-22 **(f**), and nuclear lysates were prepared and subjected to western blot analysis p65 **(g**). Nuclear histone H3 served as a loading control. * indicates *p* < 0.05

The signaling pathway(s), transcription factor(s), and DNA methylation that regulate miR-22 expression in fulvestrant-resistant 182^R^-6 cells are unknown; therefore, our next objective was to determine the molecular mechanism underlying the response of miR-22 transcription in 182^R^-6 cells to fulvestrant. Global DNA methylation profiling did not show a differential methylation in the miR-22 promoter between the parental S05 line and fulvestrant-resistant 182^R^-6 line, which suggested that DNA methylation might not be involved in miR-22 transcription. Western blot analysis indicated upregulation of phospho-HER2/neu (Tyr877), phospho-p65 (Ser276), phospho-p65 (Ser311), p50, and p65, but downregulation of phospho-p65 (Ser536) in 182^R^-6 cells (Fig. 5a). These responses correlated either positively or negatively with miR-22 expression (Fig. 1a), thereby implicating a role for HER2/NF-κB signaling in miR-22 expression in this fulvestrant-resistant cell line. We tested this hypothesis by knockdown of HER2/neu using pooled siRNAs. Western blot analysis showed that the HER2 siRNAs inhibited HER2 expression, which then led to upregulation of phospho-p56 (Ser536) and attenuation of miR-22 expression (Fig. 5b, c). An inverse correlation was observed between HER2 expression and p65 Ser536 phosphorylation, as well as between p65 Ser536 phosphorylation and miR-22 expression, suggesting an inhibitory role of p65 Ser536 phosphorylation in miR-22 transcription.

We then analyzed transcription factor binding sites in the miR-22 promoter. Bioinformatics analysis identified four potential NF-κB-binding sites (−1359/−1350, −1153/−1144, −772/−763, and −473/−464) in the regulatory region of the miR-22 gene (Fig. 5d). As expected, luciferase assays showed a significant and dose-dependent induction of luciferase activity following mutation of four potential NF-κB binding sites, and identified −1359/−1350 and −473/−464 as the two binding sites that mediate the suppressive role of phospho-p65 (Ser536) in miR-22 transcription (Fig. 5e).

No commercial ChIP-grade antibody specific to phospho-p65 (Ser536) is currently available, so we were unable to perform ChIP-PCR in this study. However, lipopolysaccharide (LPS) is known to induce NF-κB activation in breast cancer cells^30,31^, so we treated 182^R^-6 cells with LPS, alone or in combination with the NF-κB inhibitor sc-514, and examined the expression of miR-22 and nuclear RelA/p65. A qRT-PCR analysis showed that LPS alone reduced miR-22 expression, but this reduction was significantly and dose-dependently abolished by sc-514 (Fig. 5f). Western blot analysis indicated that LPS alone caused an accumulation of nuclear p65, but this enrichment was attenuated by sc-514 (Fig. 5g). An inverse correlation was observed between miR-22 expression and the levels of nuclear p65, and this was supported in two (MCF-7 and HCC1806) of the four breast cancer cell lines examined (supplementary Fig. S6). Taken together, these results suggest that HER2/neu signaling suppresses p65 Ser536 phosphorylation, which leads to transcriptional activation of miR-22.

## Discussion

Since the discovery of the microRNA lin-4 in 1993^32^, as a functionally unique small non-coding RNA, miRNAs have been intensively studied and characterized as key post-transcriptional regulators that contribute to all the biologic and pathologic processes, including drug resistance. The miRNAs have been recognized for many years to function either as tumor suppressors or as oncogenes that promote cancer progression. Certain miRNAs have dual roles, serving as both tumor suppressors and oncogenes in different tissues^33^, but a single miRNA never shows a dual role in the same cell line.

To the authors’ knowledge, this study has demonstrated, for the first time, a dual role of miR-22 in overcoming fulvestrant resistance, as both decreases and increases in miR-22 sensitize fulvestrant-resistant breast cancer cells to fulvestrant by targeting *FOXP1* and *HDAC4* and constitutive acetylation of p53. The fulvestrant-resistant 182^R^-6 breast cancer cell line showed a moderate upregulation of miR-22, which could be attributed to the miRNA biogenesis enzyme AGO2 and Dicer. Inhibition of miR-22 with an inhibitor suppressed 182^R^-6 cell proliferation, induced apoptosis, caused cell cycle S-phase arrest and enhanced the expression of p21^Cip1/Waf1^ and p27^Kip1^ (Fig. 2). Surprisingly, enforced overexpression of miR-22 also suppressed cell proliferation, induced apoptosis, caused S-phase arrest and promoted the expression of p21^Cip1/Waf1^ and p27^Kip1^ (Fig. 3). Overexpression of p21^Cip1/Waf1^ and p27^Kip1^, triggered by aberrantly expressed miR-22, may contribute to apoptosis induction and S-phase arrest^34–37^, thereby suppressing the proliferation of fulvestrant-resistant breast cancer cells. Notably, this is the first report to indicate a dual role for miR-22 in a single fulvestrant-resistant breast cancer cell line, although miR-22 has previously been reported to show contradictory functions (both oncogene and tumor suppressor functions) in the same tissue from human malignancies, including leukemia^38,39^, hepatocellular carcinoma^40,41^, and breast^21,22^ and prostate^42,43^ cancers.

Both p21 and p27, which are members of the Cip/Kip family of cyclin-dependent kinase inhibitors^34^, are well-characterized negative regulators of cell cycle. A large body of evidence has demonstrated an importance of p21 and p27 in cell cycle regulation, but other biological functions for these inhibitors have also been indicated, including apoptosis, cell fate determination, transcriptional regulation, cytoskeletal dynamics, and cell migration^44^. Consequently, aberrant expression of p21 and p27 has also been linked to breast tumorigenesis and to fulvestrant sensitivity and resistance^45–47^. Antiestrogen drugs, like fulvestrant and tamoxifen, induce cell cycle arrest of breast cancer MCF-7 cells via upregulation of p21 and p27 expression^46^. However, the breast cancer cells with acquired fulvestrant resistance, such as the 182^R^-6 cell line studied here, show p21 expression that no longer depends on either estrogens or antiestrogens^47^. The data presented here indicate that the expression of p21 is in fact downregulated in fulvestrant-resistant 182^R^-6 breast cancer cells (supplementary Fig. S2), in agreement with a previous report^47^, and this downregulation may contribute to the lack of sensitivity of these breast cancer cells to fulvestrant.

Many targets of miR-22, including p21, have been identified in the last 10 years, confirming the importance of miR-22 in tumorigenesis^48^. However, the system revealed in the present study would appear to differ from this previously reported one (supplementary Fig. S3). We examined HDAC4 and FOXP1, two other experimentally validated targets of miR-22^49,50^, to further explore the mechanism underlying the upregulation of p21 induced by miR-22 inhibition (Fig. 2e). HDAC4, as a member of class II histone deacetylases, has been implicated in tumorigenesis via transcriptional silencing of p21^27,28^. Current evidence also supports a suppressor role for the transcription factor FOXP1 in the expression of both p21 and p27 in hematopoietic stem and leukemia cells^25^. Interestingly, a recent study indicated that the majority of invasive breast cancers (67%, *n* = 133) showed a nuclear immunoreactivity of the estrogen-inducible FOXP1^51^, whereby knockdown of FOXP1 suppressed and ectopic expression promoted breast cancer cell proliferation. FOXP1 also enhanced the migration of MDA-MB-231 metastatic breast cancer cells via transcriptional repressing of NFAT1^52^. The evidence presented in the current study indicated a negative regulation of HDAC4 and FOXP1 by miR-22 in fulvestrant-resistant breast cancer cells, which supports previous reports showing that HDAC4 and FOXP1 were direct targets of miR-22^49,50^.

**Fig. 6:**
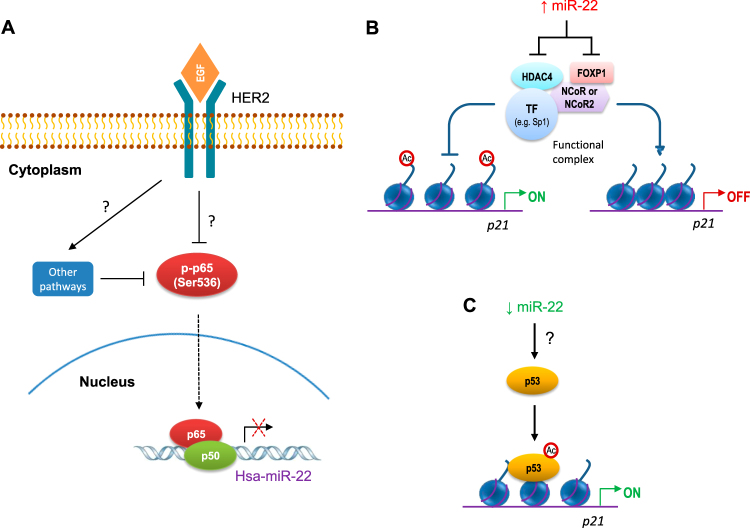
HER2/NF-κB (p65)/miR-22/HDAC4/p21 and HER2/NF-κB (p65)/miR-22/Ac-p53/p21 signaling circuits contribute to the dual role of miR-22 in resensitizing fulvestrant-resistant breast cancer cells to fulvestrant by transactivation of p21. **a** The HER2/p65 dephosphorylation (ser536) axis negatively modulates miR-22 transcription. **b** Gain-of-function of miR-22 represses the expression of HDAC4 and FOXP1, which leads to attenuation of the transcription repressor complex^63^, resulting in a transcriptional activation of p21. **c** Loss-of-function of miR-22 induces acetylation of p53 at Lys382, resulting in a transactivation of p21

Downregulation of HDAC4, induced by enforced overexpression of miR-22 in 182^R^-6 cells, resulted in an elevation of acetylated histones, including Ac-H3K9, Ac-H4K8, Ac-H4K16, and p53 acetylated at Lys382 (Fig. 4c). This increased acetylation may contribute to the upregulation of p21 and/or p27 induced by miR-22 overexpression (Figs. 3e and 6b). Furthermore, ectopic overexpression of miR-22 attenuated the expression of the transcriptional repressor complex composed of HDAC4, FOXP and Sp1 (Fig. 4d). Inhibition of miR-22 expression led to an elevation of HDAC4 and FOXP1 expression in 182^R^-6 cells (Fig. 4c, d), but it had no effect on the levels of the acetylated histones examined (Fig. 4c). This inhibition also did not result in any enrichment of the transcription factor Sp1 in the complex, as determined by immunoprecipitation (Fig. 4d), suggesting that the elevated levels of HDAC4 and FOXP1 did not effectively form a transcriptional repressor complex.

The level of p53 acetylated at Lys382 was increased in 182^R^-6 cells by miR-22 inhibition (Fig. 4c). The mechanism by which miR-22 inhibition enhanced the acetylation of p53 is unclear, but miR-22 appears to be involved in p21 transcription (Fig. 6c), in agreement with several lines of evidence from previous work^53^. This study, however, is the first report of a dual role of miR-22 in the restoration of the sensitivity to fulvestrant in a fulvestrant-resistant breast cancer cell line. Previous work has indicated that miR-22 directly targets ERα, it is downregulated in ERα + breast cancer cell lines and clinical samples^54^, and it can sensitize bladder carcinoma^55^ and resensitize paclitaxel-resistant colon cancer cells^23^ to paclitaxel. Our findings, taken together with these previous results, suggest a common theme for miR-22 in sensitizing and resensitizing certain cancer cells to chemotherapy.

In recent years, the mechanisms that control miRNA transcription have drawn much attention. A better understanding of the signaling pathways and downstream transcription factors that trigger miR-22 transcription may aid in identifying key molecules that can overcome fulvestrant resistance in breast cancer. Our data revealed a constitutive activation of HER2 by phosphorylation that resulted in a differential phosphorylation of p65 at Ser276, Ser311, and Ser536 in fulvestrant-resistant 182^R^-6 cells. The phosphorylation of p65 at Ser276 and Ser311 was strongly upregulated in fulvestrant-resistant 182^R^-6 cells (Fig. 5a), in agreement with previous reports^56^, whereas the level of phosphorylation of p65 at Ser536 was notably reduced and negatively correlated with the expression of miR-22 (Fig. 1a). Interestingly, the level of phosphorylation at Ser536 was restored by siRNA-mediated knockdown of HER2 (Fig. 5b). Concomitantly, the expression of miR-22 was reduced, implicating phosphorylation of p65 at Ser536 as a suppressive modulator of miR-22 transcription, and this was confirmed by luciferase reporter assays (Fig. 5d, e).

This study is the first to report an involvement of the activated HER2/p65 Ser536 dephosphorylation/miR-22 axis in the development of fulvestrant resistance in breast cancer (Fig. 6a). Accumulating evidence now supports the importance of the NF-κB pathway in maintaining the growth of chemoresistant breast cancers^57–59^, including the antiestrogen-resistant ones. Therefore, targeting the NF-κB pathway has been proposed as a therapeutic strategy for overcoming chemoresistance.

Breast cancer cells that have acquired resistance to chemo/radio-therapy may be more aggressive due to their metastatic potential^60^, which involves both genetic and epigenetic mechanisms. The data from the present study indicate that HER2 activation can dephosphorylate RelA/p65 at Ser536, which in turn can transactivate miR-22. An increase or a decrease in miR-22 expression can resensitize fulvestrant-resistant breast cancer cells to fulvestrant, and signaling circuits involving HER2/NF-κB (p65)/miR-22/HDAC4/p21 and HER2/NF-κB (p65)/miR-22/Ac-p53/p21 may be responsible for this dual role for miR-22 in the constitutive transactivation of p21.

## Materials and methods

### Cell culture

The MCF-7/S0.5 (S05) and MCF-7/182^R^-6 (182^R^-6) cell sublines were developed by Dr. Anne E. Lykkesfeldt (Breast Cancer Group, Cell Death and Metabolism, Danish Cancer Society Research Center, DK-2100, Copenhagen, Denmark). The ICI 182,780 (Faslodex, fulvestrant) resistant subline 182^R^-6 is derived from the parental line S05 adapted to grow in low serum (0.5%) culture medium, by long-term exposure of S05 cells to 10^–7^ M concentration of antiestrogen ICI 182,780, as described elsewhere^61^. This cell line was cultured in a DMEM/F-12 medium with 2.5 mM L-glutamine, without 4-(2-hydroxyethyl)-1-piperazineethanesulfonic acid and phenol red (HyClone), and supplemented with 1% heat-inactivated fetal bovine serum (FBS; HyClone) and 0.1 μM ICI 182,780. Human mammary epithelial cells (HMEC), purchased from Invitrogen, were cultured in a HuMEC basal serum-free medium (Invitrogen) containing HuMEC supplement (Invitrogen) and 1% penicillin/streptomycin (P/S). MCF-7 breast cancer cells were cultured in DMEM/F-12 medium (Thermo Scientific) supplemented with 10% FBS and 1% P/S. The breast cancer cell lines ZR75-1, HCC1419, and HCC1806 were purchased from ATCC and were cultured in ATCC-formulated RPMI 1640 medium (ATCC) supplemented with 10% FBS and 1% P/S. HEK293 cells were grown in DMEM/high glucose (Thermo Scientific) supplemented with 10% FBS and 1% P/S. All cell lines were incubated at 37 °C in a humidified atmosphere of 5% CO_2_. All cell lines were grown in the BSL 2 laboratory with limited personnel access; all lines are closely watched for mycoplasma and other types of contamination. No contamination or problems have been reported over the past years.

### QRT-PCR of miRNA

Total RNA, isolated from the indicated cell lines with TRIzol reagent (Invitrogen), was subjected to qRT-PCR with a primer set for hsa-miR-22 (QIAGEN) using miScript II RT Kit (QIAGEN) and QantiTect SYBR Green PCR Master Mix (QIAGEN), according to the manufacturer’s instructions. Human RNU6-2 (QIAGEN) served as the loading control. All qRT-PCR experiments were performed in triplicate and the data were analyzed using the comparative Ct method. The results were shown as a fold induction of miR-22.

### Immunofluorescence

S05 and 182^R^-6 cells were cultured on glass coverslips for 24 h (60–75% confluency), fixed with 4% paraformaldehyde in phosphate-buffered saline (PBS) and permeabilized with ice-cold methanol. After a 1 h incubation in blocking buffer, the cells were incubated in a 1:100 dilution of either anti-AGO2 or anti-Dicer monoclonal antibody in blocking buffer at 4 °C overnight. After five washes in blocking buffer, the cells were incubated with a 1:500 dilution of a 488-fluorescent conjugated secondary antibody for 2 h at room temperature and counterstained with Prolong gold mounting media (Molecular Probe). Fluorescence was observed using a confocal microscope on both the green (Alexa 488) and blue (4′,6-diamidino-2-phenylindole; DAPI) channels and analyzed in a blinded manner.

### Western blot analysis

Cells grown to ~ 95% confluence were rinsed twice with ice-cold PBS and scraped off the plate into radioimmunoprecipitation assay buffer. The proteins (30–100 µg per sample) were electrophoresed in 10% sodium dodecyl sulfate polyacrylamide gel electrophoresis and electrophoretically transferred to polyvinylidene difluoride membranes (Amersham Hybond^TM^-P, GE Healthcare) at 4 °C for 1.5 h. The blots were incubated for 1 h with 5% nonfat dry milk to block the nonspecific binding sites and subsequently incubated at 4 °C overnight with 1:200 to 1:1000 dilutions of polyclonal/monoclonal antibodies against BAX (Cat# sc-7480), Bcl2 (Cat# sc-7382), p27 (Cat# sc-56338), p53 (Cat# sc-56182), p-p65 (Ser276, Cat# sc-101749), p-p65 (Ser311, Cat# sc-33039) and Sp1 (Cat# sc-420) (all from Santa Cruz Biotechnology) or CDK2 (Cat# 2546), CDK6 (Cat# 3136), cyclin D1 (Cat# 2922), cyclin E1 (Cat# 4129), Drosha (Cat# 3364), FOXP1 (Cat# 2005), HDAC4 (Cat# 15164), HER2 (Cat# 2248), NF-κB1 p105/p50 (Cat# 13586), NF-κB p65 (Cat# 4764), phospho-HER2 (Tyr877, Cat# 2241) and phosphor-p65 (Ser536, Cat# 3031) (all from Cell Signaling Technology); or AGO2 (Cat# ab32381), Dicer (Cat# ab14601), ERα (Cat# ab16460), histone H3 (Cat# ab1791), and p21 (Cat# ab7960) (all from Abcam). Immunoreactivity was detected using a peroxidase-conjugated antibody and visualized with the ECL Plus Western Blotting Detection System (GE Healthcare). The blots were stripped before reprobing with antibodies against actin (Abcam, Cat# ab179467) or GAPDH (Santa Cruz Biotechnology, Cat# sc-47724).

### Knockdown of HER2/Neu

182^R^-6 cells grown to 80% confluency were transiently transfected with either 100 nM Neu siRNA (pool of 3–5 target-specific sequences 19–25 nucleotides in length; Santa Cruz Biotechnology) or control siRNA-A (Santa Cruz Biotechnology) using Lipofectamine 3000 (Invitrogen) as per the manufacturer’s instructions. At 72 h after transfection, whole cell lysates were prepared for western blot analysis.

### Bioinformatics

Transcription factor NF-κB/p65 binding sites in the hsa-miR-22 promoter were analyzed using TFBIND software. Interactions between miR-22 and the 3′-UTRs of p21, HDAC4 and FOXP1 mRNAs were analyzed using RNAHybrid and TargetScan.

### Generation of a wild-type hsa-miR-22 promoter reporter plasmid

The *Homo sapiens* miR-22 stem loop (accession: MI0000078), which contains hsa-miR-22-3p and hsa-miR-22-5p, is located in a non-protein-coding gene MGC14376 (MIR22HG-001 ENST00000334146, Ensembl) exon 3, chromosome 17. A 1547 bp DNA fragment, containing a 1404 bp promoter, a 81 bp exon 1 and a 62 bp intron 1 of the miR-22 host gene MGC14376, was amplified by genomic PCR, cloned to the pGEM-T easy vector (Promega), released by restriction digestion with FastDigest *Kpn*I and *Hin*dIII (Fermentas), and subcloned into the pGL3-Basic vector (Promega) to generate the WT-miR-22 Prom reporter. Sequence identity of the insert fragment was confirmed by automatic DNA sequencing. Primers used for amplifying hsa-miR-22 promoter were the following: forward primer 5′-ATGGTACCGAGGTCACACTTTC-3′ and reverse primer 5′-TTAAGCTTTCACCCTCCATCC-3′.

### Site-directed mutagenesis

Site-directed mutation of putative NF-κB/p65-binding motifs in the hss-miR-22 promoter was carried out using the QickChange II Mutagenesis Kit (Agilent Technologies), as per the manufacturer’s instructions. The following primers were utilized to generate site-directed mutants: NF-kB MT1-F: 5′-GCTGTCGCCCTTTAAGGAAAACCCCGGGCTGGATTCCG-3′, NF-kB MT1-R: 5′-CGGAATCCAGCCCGGGGTTTTCCTTAAAGGGCGACAGC-3′; NF-kB MT2-F: 5′-CTTGGGAGGCTCTGGGGGAAATTCTGGGACTGGGAAGAG-3′, NF-kB MT2-R: 5′-CTCTTCCCAGTCCCAGAATTTCCCCCAGAGCCTCCCAAG-3′; NF-kB MT3-F: 5′-CCACATGTTCCTCTTTGGGGGAAAAGCGGGGATTGATAAGGTAGG-3′, NF-kB MT3-R: 5′-CCTACCTTATCAATCCCCGCTTTTCCCCCAAAGAGGAACATGTGG-3′; NF-kB MT4-F: 5′-CTGGGGCGCAAGGCAGGAAAAATCCTTAAAGGCGCAATGTCC-3′ and NF-kB MT4-R: 5′-GGACATTGCGCCTTTAAGGATTTTTCCTGCCTTGCGCCCCAG-3′. All mutant constructs were confirmed by automated DNA sequencing.

### Construction of a p21 luciferase reporter

A p21 luciferase reporter bearing either a predicted wild-type or mutant miR-22 binding site was generated by synthesizing the following oligos: *p21* WT-3′UTR1: 5′-/5Phos/CTAGACTAGTTCTACCTCAGGCAGCTG-3′, *p21* WT-3′UTR2: 5′-/5Phos/AATTCGAGCTGCCTGAGGTAGAACTAGT-3′; *p21* MT3′UTR1: 5′-/5Phos/CTAGACTAAAAATACCTCAGAAAAATCG-3′ and *p21* MT3′UTR2: 5′-/5Phos/AATTCGATTTTTCTGAGGTATTTTTAGT-3′. After annealing, the double-stranded oligos were cloned downstream of the luciferase gene in the pGL3-Basic vector, between *Xba*I and *EcoR*I (a linker introduced by Mr. James Meservy) to generate Luc-WT-p21-3′UTR and Luc-MT-p21-3′UTR reporters. The sequence identity was confirmed by automated DNA sequencing. The underline indicates the mutated deoxy nucleotide.

### Luciferase assay

HEK293 cells grown to 90% confluency in six-well plates were transiently cotransfected with either 50 ng or 100 ng reporter plasmid (either WT-miR-22 Prom or MT-NF-κB1-4), 0.5 μg GFP-RelA (a RelA/p65 expression plasmid that was a gift from Warner Greene, Addgene plasmid # 23255; see ref 62), and 5 ng pRL-TK plasmid (Promega); or they were cotransfected with 0.5 μg of either WT-p21-3′UTR or MT-p21-3′UTR luciferase reporter and 5 ng of pRL-TK plasmid in combination with the indicated concentration of hsa-miR-22 mimic using Lipofectamine 3000 (Invitrogen) per manufacturer’s instruction. At 24 h after transfection, the cells were lysed in passive lysis buffer and the relative luciferase activity was determined by the Dual-Luciferase Reporter Assay System (Promega) using a luminometer (FLUOstar Omega, BMG LABTECH) with Firefly luciferase data normalized to Renilla. Two independent assays were repeated, each was done in duplicate.

### Inhibition of NF-κB activity

182^R^-6 cells grown to 80% confluency were exposed to the indicated concentrations of sc-514, the IKK2 inhibitor. After 1 h of exposure, the cells were incubated with the indicated concentration of LPS. 24 h after treatment, total RNA was isolated using TRIzol reagent (Invitrogen) and nuclear lysates were prepared using NE-PER^TM^ Nuclear and Cytoplasmic Extraction Reagents (Pierce), following the manufacturers’ instructions.

### MTT assay

182^R^-6 cells grown to 80% confluency were transfected with either 50 nM miR-22 inhibitor or negative control A (all purchased from Exiqon), or 40 nM miR-22 mimic or AllStars negative control siRNA (all purchased from QIAGEN). 24 h after transfection, 3.0 × 10^3^ cells were replated in 96-well plates. Assays using 3-(4,5-Dimethylthiazol-2-yl)-2,5-diphenyltetrazolium bromide (MTT) assays were performed using the Cell Proliferation Kit I (Roche Diagnostics GmbH) in triplicate, as described by the manufacturer. The spectrophotometric absorbance of samples was measured at 595 nm using a microtiter plate reader (FLUOstar Omega, BMG LABTECH).

### Cell cycle and apoptosis analyses

182^R^-6 cells grown to 80% confluency were transfected with either 50 nM miR-22 inhibitor or negative control A (all purchased from Exiqon), or 40 nM miR-22 mimic or AllStars negative control siRNA (all purchased from QIAGEN). At 72 h or 96 h after transfection, the cells were harvested for cell cycle and apoptosis analyses, which were carried out with a BD FACSCanto^TM^ II Flow Cytometer (BD Biosciences) using a propidium iodide staining solution and a BD Pharmingen^TM^ V-FITC Annexin Apoptosis Detection Kit (BD Biosciences) in triplicate, according to the manufacturers’ instructions.

### Statistical analysis

The Student’s *t* test was used to determine the statistical significance of differences between groups in hsa-miR-22 expression, cell growth, cell cycle, apoptosis, and luciferase activity. A value of *p* < 0.05 was considered statistically significant.

## Electronic supplementary material

Supplementary figure captions

Suplementary figures
