# Supplementary material for: A dual role of miR-22 modulated by RelA/p65 in resensitizing fulvestrant-resistant breast cancer cells to fulvestrant by targeting FOXP1 and HDAC4 and constitutive acetylation of p53 at Lys382
Source: Oncogenesis. 2018 Jul 30;7(7):54. doi: 10.1038/s41389-018-0063-5 (PMC6064715; doi:10.1038/s41389-018-0063-5)
Supplement: Supplementary file 1 — Supplementary figure captions [file 41389_2018_63_MOESM1_ESM.docx]

**SUPPLEMENTARY FIGURES**

Supplementary **Fig. S1** Differential expression of miRNAs with identical alteration in both 182^R^-6 and TAM^R^-1 cell lines. miRNA microarray and data analysis were carried out as described in “Materials and methods”. **a** Venn diagram showing a number of differentially expressed miRNAs identified by microarray. The differentially expressed miRNAs with identical alteration in both 182R-6 and TMAR-1 cell lines were listed in table S1.

Supplementary **Fig. S2** Downregulation of p21 in fulvestrant-resistant 182^R^-6 cells. **a** and **b** Whole cell lysates prepared from S05 and 182R-6 cells were subjected to western blot analysis using antibodies to ERα, p21, p27, CDK2, CDK6, and cyclin D1. Actin served as a loading control.

Supplementary **Fig. S3** p21 may not be a direct target of miR-22. **a** Diagram of the predicted “seed” of miR-22 targeting the 3’UTR of p21 mRNA. **b** HEK293 cells grown to 90% confluency were transiently cotransfected with 0.5 μg of either WT-p21-3’UTR or MT-p21-3’UTR luciferase reporter, 5 ng of pRL-TK plasmid in combination with the indicated concentration of hsa-miR-22 mimic. At 24 h after transfection, the luciferase activity was measured as described in the “Methods” section.

Supplementary **Fig. S4** The highly conserved miR-22 targeting sites in the 3’UTR of FOXP1 and HDAC4 mRNAs. **a** and **b** miR-22 targeting sites were predicted for FOXP1 (a) and HDAC4 (b) mRNAs in different species.

Supplementary **Fig. S5** TET2 may not be a direct target of miR-22. 182^R^-6 cells transfected with either 40 nM miR-22 mimic or AllStars negative control siRNA or 50 nM miR-22 inhibitor or negative control were incubated for either 72 or 96 h, whole cell lysates were prepared and subjected to western blot analysis using antibody to TET2. GAPDH served as a loading control.

Supplementary **Fig. S6** Correlation between HER2 activation and RelA/p65 phosphorylation at Ser536. **a** Whole cell lysates prepared from HMEC, MCF7, ZR75-1, HCC1419 and HCC1806 cells were subjected to western blot analysis using the indicated antibodies, as described in the “Methods” section. GAPDH served as a loading control. **b** Total RNA isolated from HMEC, MCF7, ZR75-1, HCC1419 and HCC1806 cells was subjected to qRT-PCR analysis using a primer set of hsa-miR-22, as described in the “Methods” section. RNU6-2 was used as a reference gene to normalize the miR-22 expression. ** indicates p<0.001.
