# Supplementary material for: A dual role of miR-22 modulated by RelA/p65 in resensitizing fulvestrant-resistant breast cancer cells to fulvestrant by targeting FOXP1 and HDAC4 and constitutive acetylation of p53 at Lys382
Source: Oncogenesis. 2018 Jul 30;7(7):54. doi: 10.1038/s41389-018-0063-5 (PMC6064715; doi:10.1038/s41389-018-0063-5)
Supplement: Supplementary file 2 — Suplementary figures [file 41389_2018_63_MOESM2_ESM.pptx]

## Slide 1
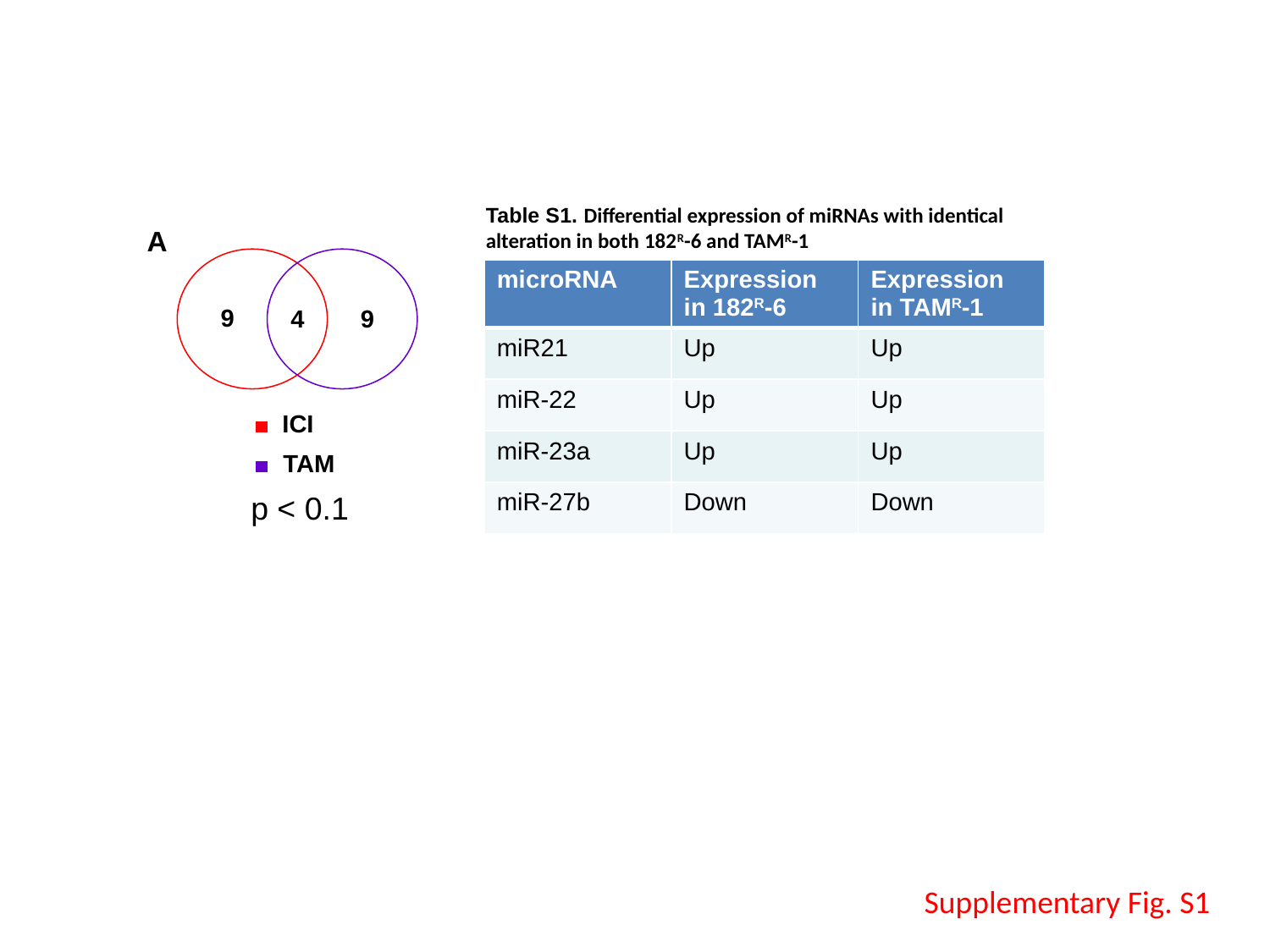

Table S1. Differential expression of miRNAs with identical alteration in both 182R-6 and TAMR-1
A
| microRNA | Expression in 182R-6 | Expression in TAMR-1 |
| --- | --- | --- |
| miR21 | Up | Up |
| miR-22 | Up | Up |
| miR-23a | Up | Up |
| miR-27b | Down | Down |
9
4
9
ICI
TAM
p < 0.1
Supplementary Fig. S1

## Slide 2
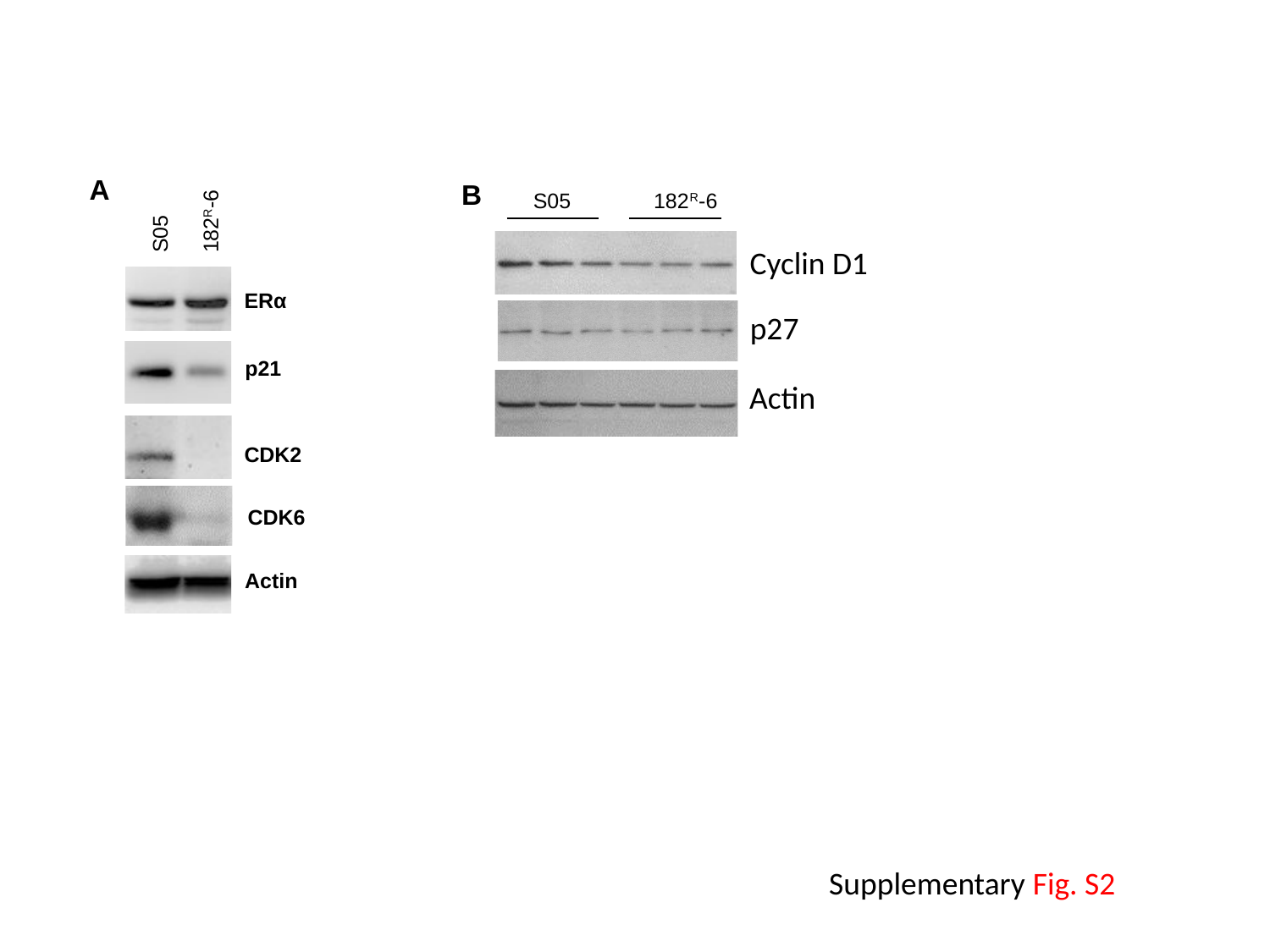

S05
182R-6
A
B
S05 182R-6
Cyclin D1
ERα
p27
p21
Actin
CDK2
CDK6
Actin
Supplementary Fig. S2

## Slide 3
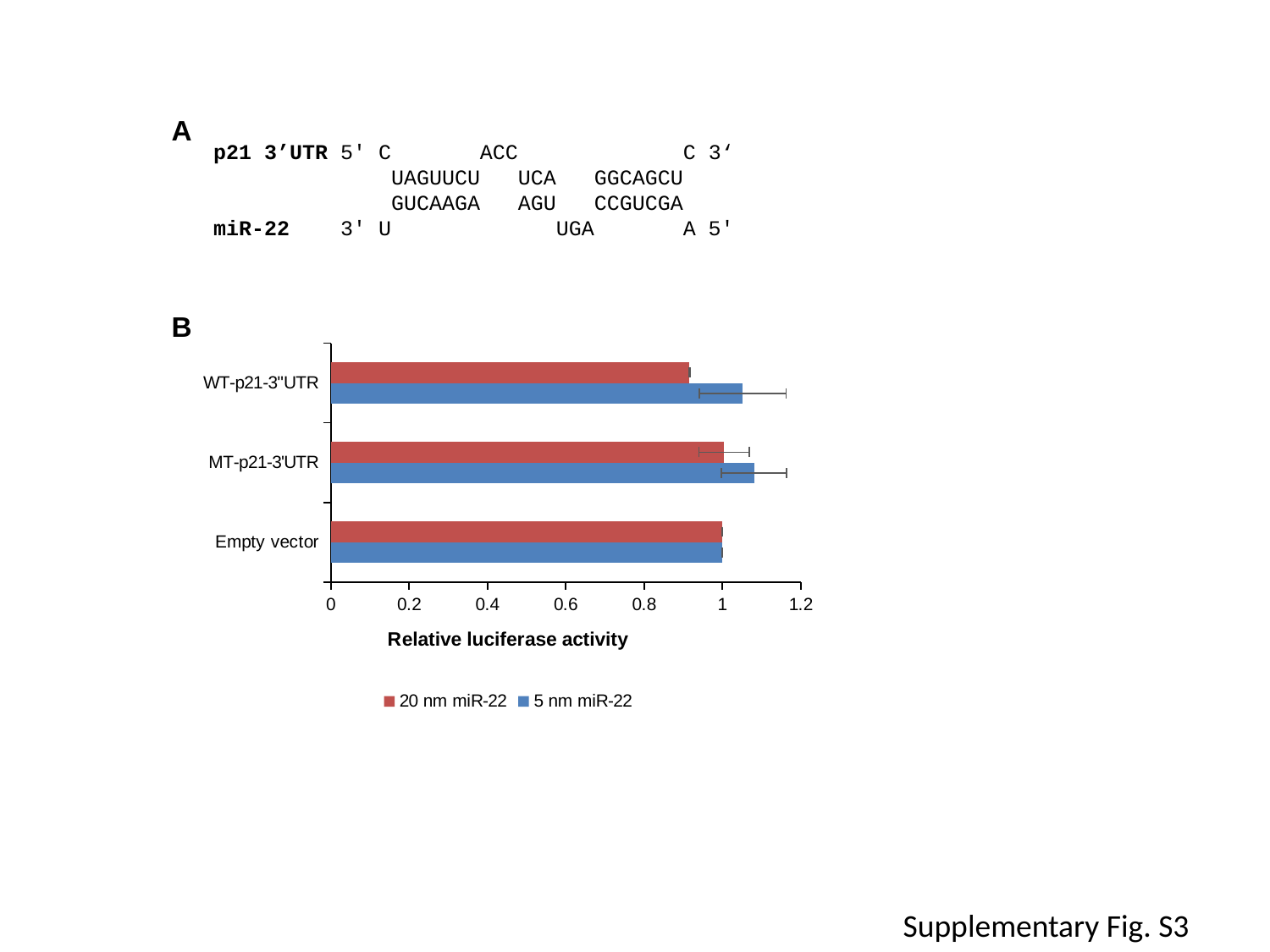

A
p21 3’UTR 5' C ACC C 3‘
 UAGUUCU UCA GGCAGCU
 GUCAAGA AGU CCGUCGA
miR-22 3' U UGA A 5'
B
### Chart
| Category | 5 nm miR-22 | 20 nm miR-22 |
|---|---|---|
| Empty vector | 1.0 | 1.0 |
| MT-p21-3'UTR | 1.0809530569808647 | 1.0046916276257698 |
| WT-p21-3"UTR | 1.051953168387553 | 0.9156240711092531 |Supplementary Fig. S3

## Slide 4
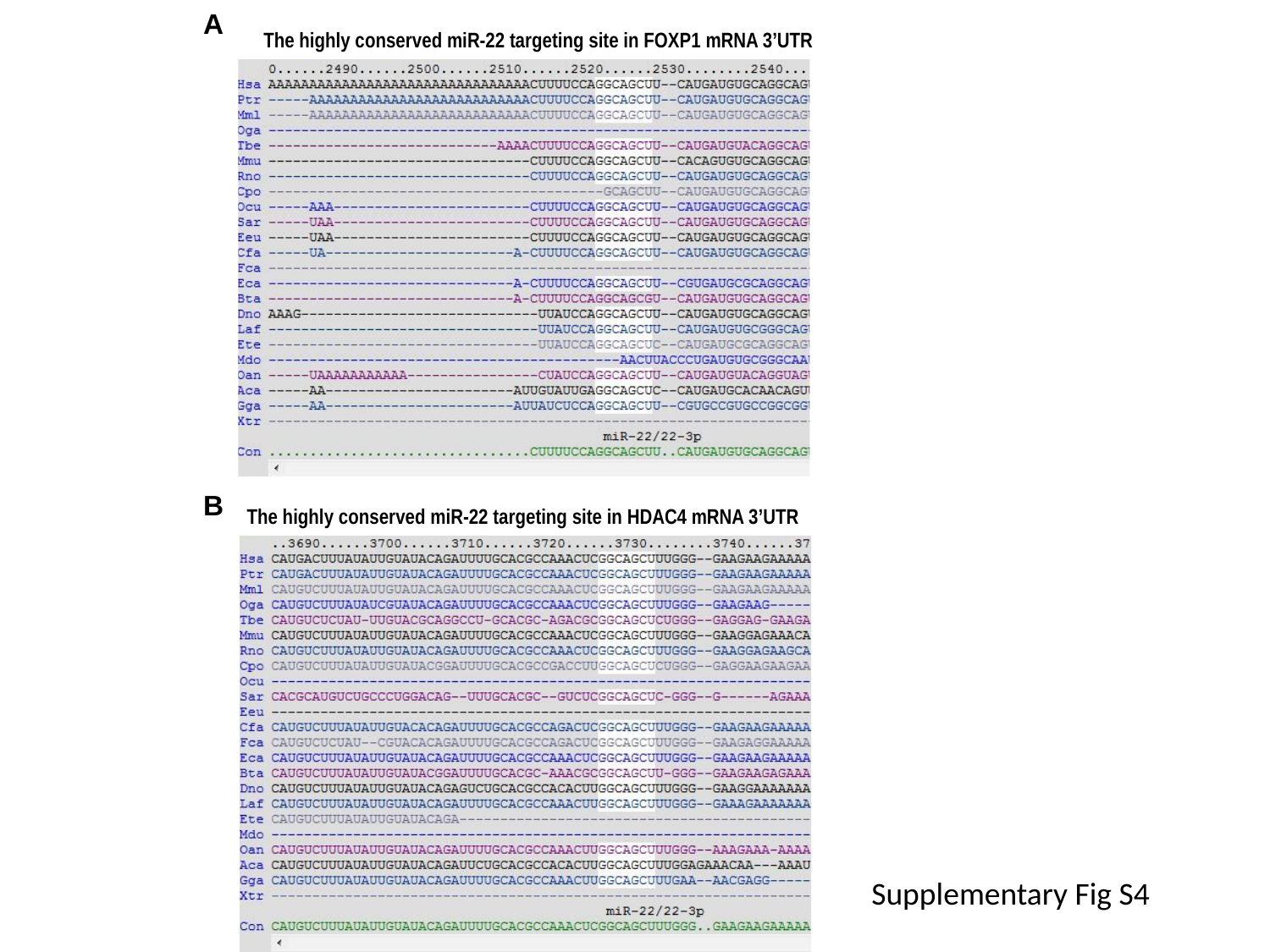

A
The highly conserved miR-22 targeting site in FOXP1 mRNA 3’UTR
B
The highly conserved miR-22 targeting site in HDAC4 mRNA 3’UTR
Supplementary Fig S4

## Slide 5
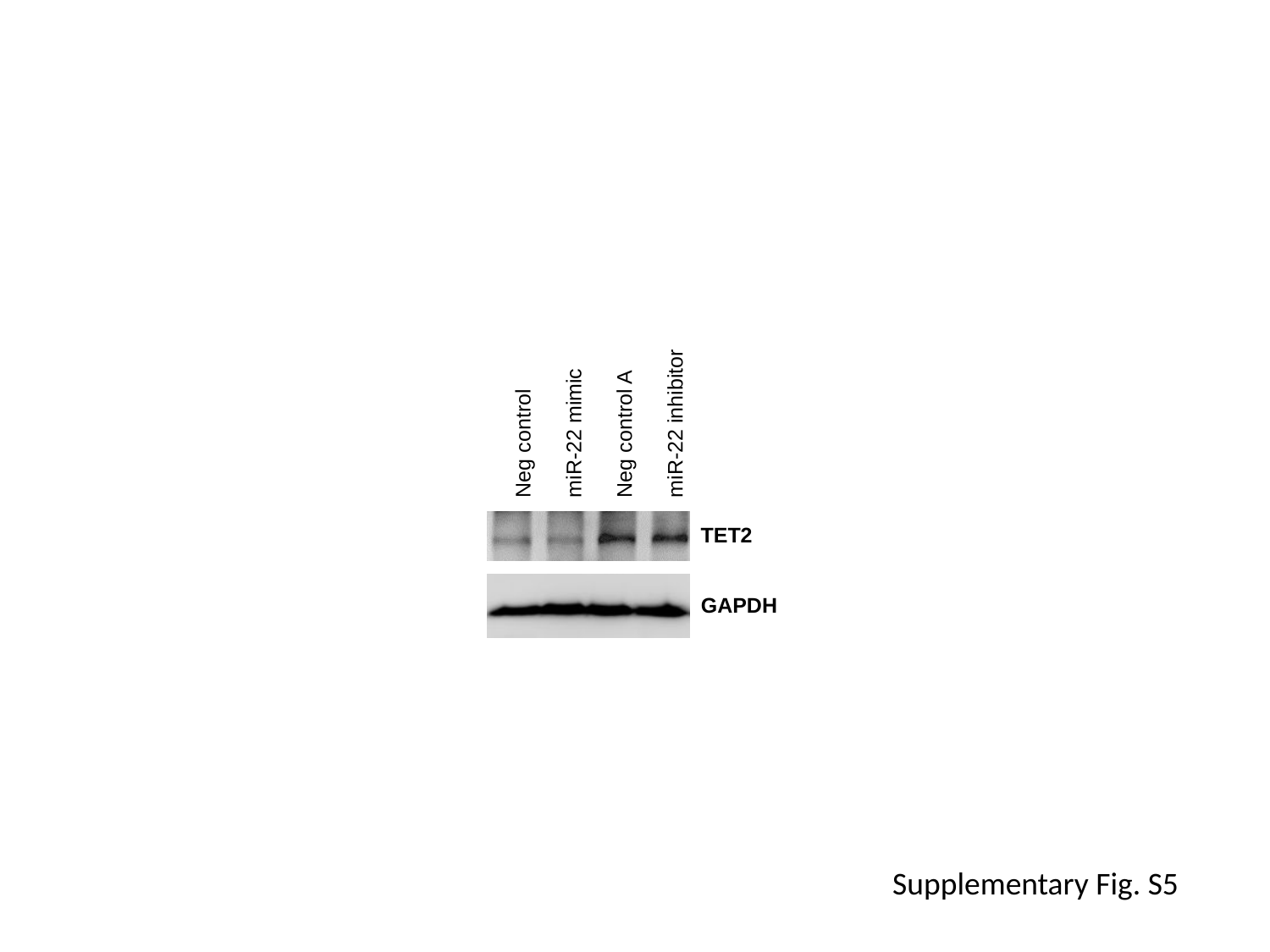

Neg control
miR-22 mimic
Neg control A
miR-22 inhibitor
TET2
GAPDH
Supplementary Fig. S5

## Slide 6
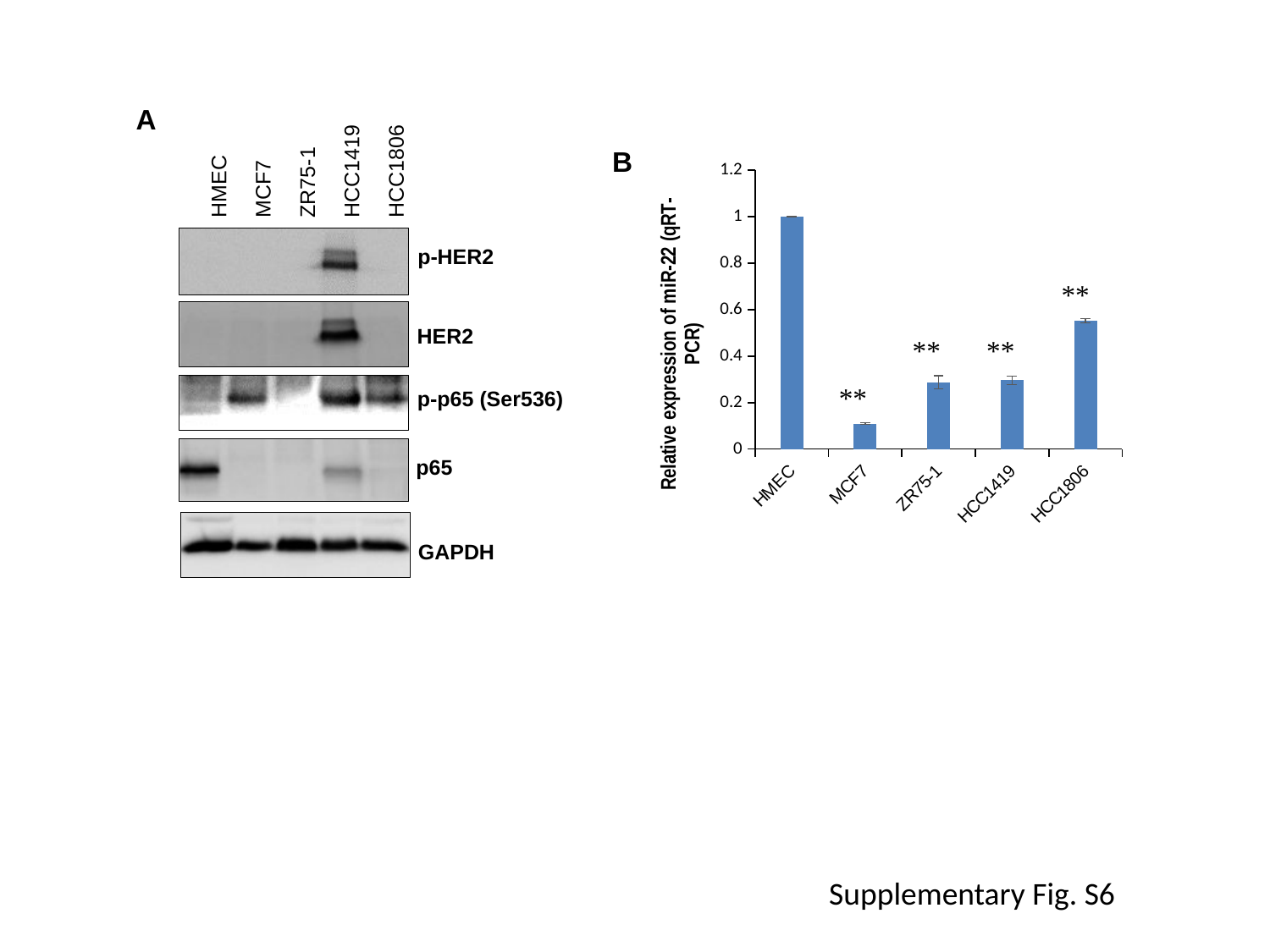

HMEC
MCF7
ZR75-1
HCC1419
HCC1806
A
B
### Chart
| Category | |
|---|---|
| HMEC | 1.0 |
| MCF7 | 0.10962514888771761 |
| ZR75-1 | 0.2874138735223742 |
| HCC1419 | 0.2956037011347147 |
| HCC1806 | 0.5522830771819135 |
p-HER2
**
HER2
**
**
**
p-p65 (Ser536)
p65
GAPDH
Supplementary Fig. S6
